# Psychophysical Evaluation of the Capability for Phantom Limb Movement in Forearm Amputees

**DOI:** 10.1371/journal.pone.0156349

**Published:** 2016-05-26

**Authors:** Noritaka Kawashima, Tomoki Mita

**Affiliations:** Research Institute, National Rehabilitation Center for Persons with Disabilities, Tokorozawa, Saitama, Japan; University of Waterloo, CANADA

## Abstract

A phantom limb is the sensation that an amputated limb is still attached to the body and is moving together with other body parts. Phantom limb phenomenon is often described on the basis of the patient’s subjective sense, for example as represented using a visual analog scale (VAS). The aim of this study was to propose a novel quantification method for behavioral aspect of phantom limb by psychophysics. Twelve unilateral forearm amputees were asked to perform phantom wrist motion with various motion frequencies (60, 80, 100, 120, 140, 160, 180, 200, 220, 240% of preferred speed). The attainment of phantom limb motion in each session was rated by the VAS ranging from 0 (hard) to 10 (easy). The relationship between the VAS and motion frequency was mathematically fitted by quadric function, and the value of shift and the degree of steepness were obtained as evaluation variables for the phantom limb movement. In order to test whether the proposed method can reasonably quantify the characteristics of phantom limb motion, we compared the variables among three different phantom limb movement conditions: (1) unilateral (phantom only), (2) bimanual, and (3) bimanual wrist movement with mirror reflection-induced visual feedback (MVF). While VAS rating showed a larger extent of inter- and intra-subject variability, the relationship of the VAS in response to motion frequency could be fitted by quadric curve, and the obtained parameters based on quadric function well characterize task-dependent changes in phantom limb movement. The present results suggest the potential usefulness of psychophysical evaluation as a validate assessment tool of phantom limb condition.

## Introduction

Most amputees still have a sensation and perception of the missing part even after limb amputation. This phenomenon is widely known as “phantom limb” [1,2]. While some amputees have vivid kinesthesia for their phantom limb, previous studies have described others as having an awareness of the missing limb as clenched and paralyzed in a specific position [3–6]. The literature indicates that there is a large inter-individual difference in the modality and extent of phantom limb awareness. There are many elements that affect the phantom limb condition, for example, missing part of body, type of prosthesis, pre-amputation condition, etc. Therefore, it is quite difficult to characterize the phantom limb phenomenon. A traditional and conventional way to characterize phantom limb is by describing the patient’s subjective feeling as a case report and presenting case series. Almost all outcome measures employed in behavioral and clinical studies regarding the extent of awareness and condition of a phantom limb were subjective or analyzed on a psychological scale, such as the visual analog scale (VAS).

It has been recognized that cortical reorganization at least partly explains phantom limb sensation and pain with the concept of maladaptive plasticity [7–12]. On the other hand, recent findings suggested an evidence of the inverse to maladaptive plasticity, that is, reorganization related to sensory deprivation and preserved function associated with pain [13–15]. While cortical mechanisms underlying phantom limb remain a matter of debate, it is important to establish a reliable method to characterize phantom limb phenomenon. To the best of our knowledge, there are not studies that quantify the condition, situation and/or state of phantom limb phenomenon itself.

The purpose of the present study was therefore to propose a novel quantification method by psychophysical evaluation for the behavioral and phenomenological aspect of phantom limb. We asked forearm amputees to conduct phantom wrist flexion-extension movement at nine different tempos of a metronome. Immediately following 30 seconds of movement, the subjects were asked to rate the degree of attainment of phantom wrist motion using the VAS. We attempted to adopt a mathematical fitting as VAS changes in response to variations in motion frequency. We assumed that if the subjects’ self report (subjective scale) correctly reflects the attainment of phantom limb movement, the VAS might change in accordance with motion frequency, and could thus be characterized by psychophysical profile.

In order to test whether the proposed method can reasonably quantify the characteristics of phantom limb movement, we compared the psychophysical profiles among following three experimental conditions: (1) unilateral (phantom only), (2) bilateral, and (3) bilateral wrist movement with mirror reflection-induced visual feedback (MVF). Since previous studies have demonstrated that MVF regarding the missing limb enhances awareness of kinesthesia in most amputee populations [3,16–18], we assume that the proposed method would suitably identify the task-dependent changes in the phantom limb movement.

## Materials and Methods

Twelve male unilateral upper limb amputees participated in the present study (age: 59.0 ± 16.59 yrs). Nine subjects had undergone unilateral forearm amputation following a work-related traumatic injury, two subjects as a result of car accidents, and one due to post-cancer surgery. The stump length (distal to proximal end of the radius) ranged from 88 to 209 mm. Each subject was interviewed by one author (M.T.). The interviews documented medical history, present residual-limb (stump) sensations and pain, condition of the phantom limb, and phantom pain. The experimental protocol of this study was approved by the research ethics boards of the National Rehabilitation Center for Persons with Disabilities (NRCD). Written informed consent was obtained from all subjects before participation in the study.

Table 1 provides the details of the subjects. Except for Subject E, the condition of the phantom limb in all subjects was categorized as “telescoping,” meaning that the phantom hand had shrunk into the stump. The different subjects used different types of prostheses in daily life. Four of 12 subjects had phantom limb pain and eight did not. For the determination of preferred speed of the phantom wrist movement, the experimenter asked subjects to attempt periodic wrist flexion-extension movement of their phantom limb at a pace that is comfortable for them. The experimenter determined the approximate rate per minute (rpm) of the preferred speed in steps of 5 rpm. The determined preferred speed and an attainment of phantom limb movement is represented in the two rightmost columns in Table 1.

**Table 1:** Patients characteristics.

	Age	Amputation side	Dominant Hand	Stump Length [mm]	Phantom limb				Cause of amputation	Daily prosthesis use	Phantom wrist motion	
					Awareness	Pain	Numbness	Telescoping			Preferred speed	VAS
Subject A	65	Left	Right	190	+	-	+	+	Trauma	Cable operated	60	10
Subject B	33	Right	Left	150	+	-	+	+	Trauma	Cable operated & cosmetic	60	8
Subject C	76	Left	Right	110	+	-	+	+	Trauma	Cable operated	55	9
Subject D	64	Right	Right	95	+	-	+	+	Trauma	Cable operated & cosmetic	80	10
Subject E	65	Right	Right	112	+	+	+	-	Trauma	Myoelectric	50	9
Subject F	33	Right	Right	125	+	+	+	+	Trauma	none	50	9
Subject G	43	Left	Right	150	+	+	+	+	Trauma	Cosmetic	55	10
Subject H	63	Right	Right	209	+	-	+	+	Trauma	Cable operated	50	9
Subject I	71	Right	Right	140	+	-	-	+	Trauma	Cable operated	55	10
Subject J	63	Left	Right	125	+	-	-	+	Tumor	Cosmetic	60	9
Subject K	51	Left	Right	88	+	-	-	+	Trauma	Myoelectric & cosmetic	70	8
Subject L	31	Right	Right	135	+	+	+	+	Trauma	Myoelectric & cosmetic	60	7

### Experimental procedures

**Fig 1:**
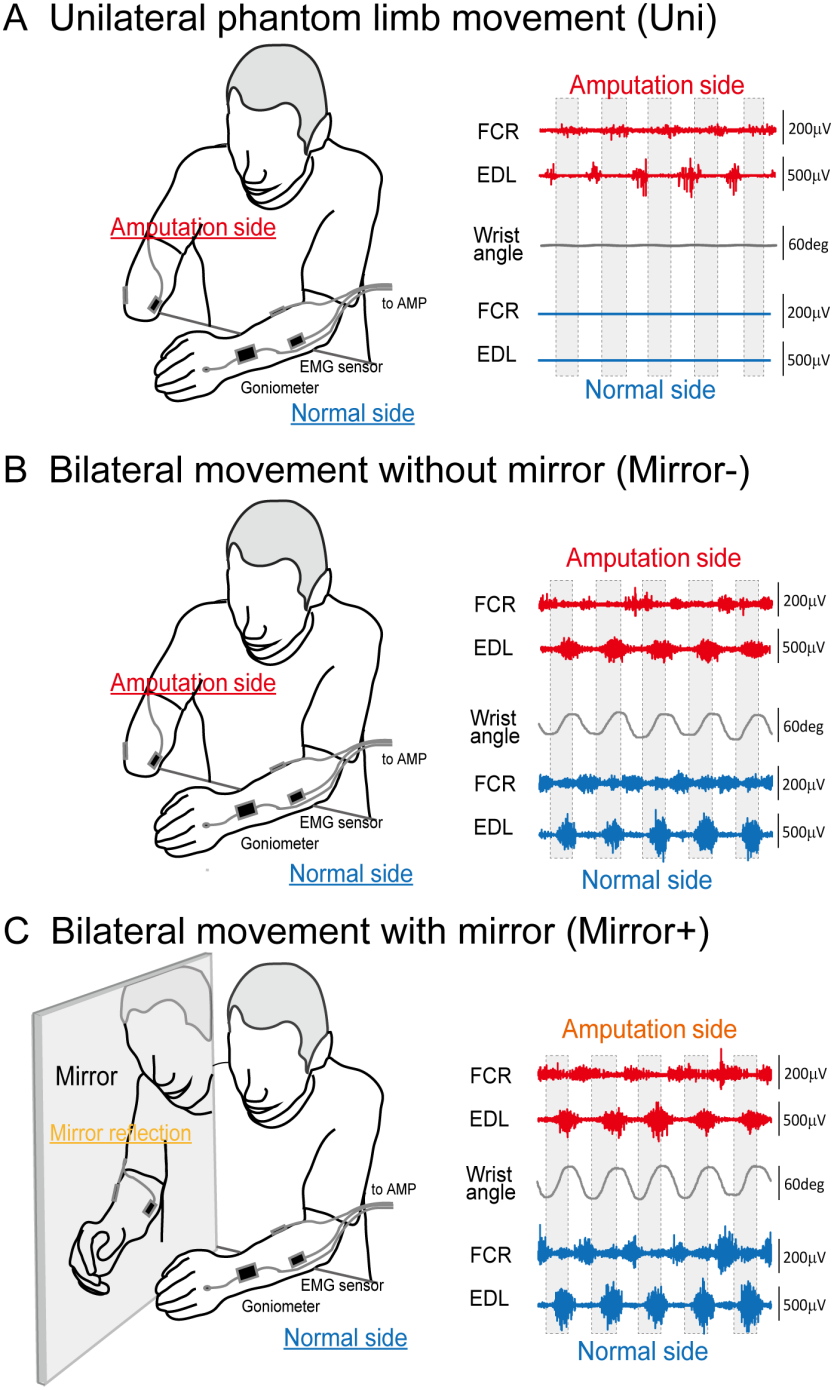
Experimental set-up. Subjects were asked to conduct wrist motion of the phantom limb in three conditions: (1) unilateral (phantom only), (2) bimanual, and (3) bimanual wrist movement with visual feedback of the phantom limb by way of a mirror reflection of the intact hand. Wrist joint angle of intact side and EMG activity of the FCR and EDL muscles in both side were recorded during ten different rhythmic wrist flexion-extension movement in each condition.

Each subject was asked to sit in a chair and place his arm on a table. The subject was then instructed to perform periodic (flexion to extension and vice versa) phantom wrist movement following three different experimental conditions: (1) unilateral (Uni; phantom only), (2) bilateral without mirror (Mirror-), and (3) bilateral with mirror (Mirror+). Under the Mirror+ condition, the intact arm was placed on one side of the mirror such that the subject could see the reflection of the intact hand as if it were the missing hand (Fig 1). The phantom wrist motion was performed at various motion frequencies (60, 80, 100, 120, 140, 160, 180, 200, 220, and 240% of preferred speed). The subjects were instructed to maintain the motion for 30 seconds in each motion frequency trial. In order to eliminate ordering effects, the order of the condition and the arrangement of the motion frequencies were randomized.

### Data recording

Immediately following completion of each trial, subjects were asked to describe the attainment of phantom limb motion using the VAS, which ranges from 0 (impossible) to 10 (motion completed). During movement, bilateral muscle electromyographic (EMG) activity was obtained from the extensor digitorum longus (EDL) and flexor carpi radialis (FCR) muscles, both of which were present in all subjects, with bipolar electrodes. The EMG signal was amplified and band-pass filtered between 20 and 450 Hz (Bagnoli-8 EMG System; Delsys, Inc., Boston, MA, USA). In order to measure changes in wrist angle, an electrogoniometer (Goniometer System; Biometrics Ltd., Ladysmith, VA, USA) was attached to the wrist joint on the intact side. Because we instructed subjects to move both wrists synchronously, we assumed that the motion of the phantom limb could be characterized by measuring the motion of the intact side. Since EMG data were obtained on different days, care should be taken in comparing these data. We used bipolar electrodes (DE-2.3; Delsys) with a constant inter-electrode distance of 1 cm, and tried to place the electrodes at the same locations for all three experiments. Furthermore, in order to reduce impedance between electrode and muscle, skin preparation (abrasion, cleaning with alcohol) was carried out carefully before recording. During the experiment, all data were continuously monitored by Power Lab software (Chart ver. 5; ADInstruments, USA) and were digitized at 1 kHz for later analysis.

### Data analysis

Based on the VAS results at different motion frequencies, we adopted a quadric curve fitting as VAS changes in response to motion frequency variation. The value of shift (motion frequency at maximum VAS) and the degree of steepness (identified as the coefficient of the squared term) were obtained as evaluation variables. In order to evaluate whether these parameters reflect the accomplishment of phantom limb motion, wrist range of motion (ROM) and the root mean squared value of stump muscle EMG were also calculated. The EMG signals were full-wave rectified after subtracting the offset level, which was detected by the averaged value of the raw EMG signal. It was then averaged over the last five motion cycles for each trial. The magnitude of EMG activity for each FCR and EDL muscle was quantified as the mean amplitude during the muscle activation period. The duration over which the muscle was active was calculated considering the muscle to be active when its averaged EMG signal consistently exceeded the level of resting EMG activity (mean value plus three times its standard deviation).

### Additional experiment

In order to test whether the repetition of the trial affected data reproducibility and validity for quadric curve fitting, we asked Subject L to participate in an additional experiment consisting of 5 series of 9 different frequencies of bilateral movement without MVF (similar to the Mirror- condition). This was to test whether the coefficient of determination changes with the repetition of the session.

### Statistical analysis

Kolmorov-Smirnoff test was used to confirm the data can be regarded as normal distribution. Statistical differences in the maximum value of the y coordinate and the slope of the quadric function among the three groups were tested by repeated one-way analysis of variance (ANOVA), and the post-hoc test (Tukey-test) was then applied for comparison among experimental conditions. Correlation coefficient was calculated to test relationship between ROM and VAS. Values were represented as mean±SD. Significance was accepted at p<0.05.

## Results

**Fig 2:**
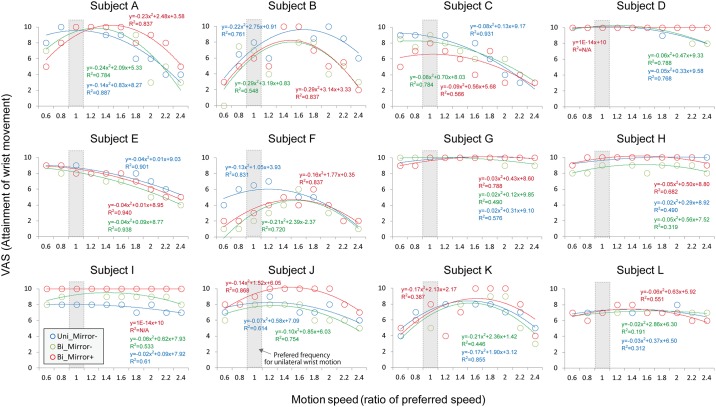
Relationship between motion frequency and an attainment of phantom limb motion as reported by VAS obtained from all subjects. VAS systematically changed with motion frequency, and showed task-dependent differences among the three experimental conditions. The quadric (inverted U-shaped) function was applied to the relationship between motion frequency and VAS.

As shown in Fig 2, the attainment of phantom limb motion as reported by VAS systematically changed with motion frequency, and showed task-dependent differences among the three experimental conditions. Although the preferred speed of the phantom limb motion was different among subjects, the VAS profile in accordance with motion frequency could be well fitted by the quadric (inverted U-shaped) function. Table 2 shows the coefficient determination (R^2^) of the quadric function in each condition obtained from all subjects. This mathematical fitting explains almost 60–70% of variance in the relationship between VAS and motion frequency (Uni: R^2^ = .71±0.91, Mirror-: R^2^ = .62±0.23, Mirror+: R^2^ = .71±0.16). Since Subjects D and G were able to accomplish phantom limb movement with the highest VAS at all frequencies under the Mirror+ condition, R^2^ value was not available for this condition for these two patients.

**Table 2:** Coefficient determination (R^2^) of the quadric function in each condition obtained from all subjects.

	Unilateral	Bilateral	
		Mirror-	Mirror+
Subject A	0.887	0.784	0.837
Subject B	0.761	0.548	0.718
Subject C	0.931	0.889	0.613
Subject D	0.768	0.788	N/A
Subject E	0.900	0.938	0.940
Subject F	0.831	0.720	0.709
Subject G	0.611	0.533	N/A
Subject H	0.491	0.319	0.682
Subject I	0.312	0.200	0.551
Subject J	0.576	0.490	0.788
Subject K	0.856	0.446	0.387
Subject L	0.614	0.754	0.868
**Average**	0.712	0.617	0.709
**SD**	0.191	0.230	0.163

**Fig 3:**
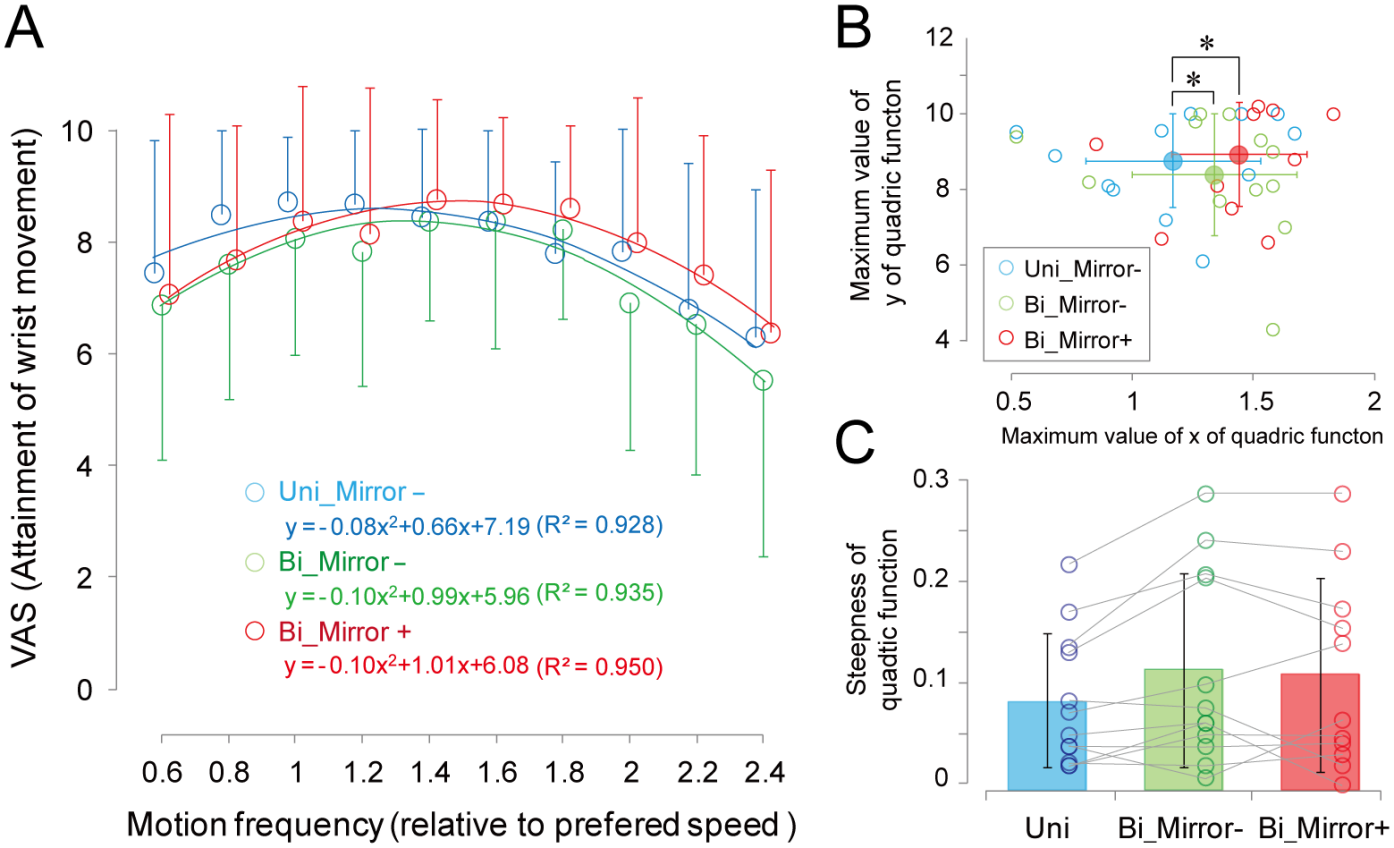
Averaged data of the VAS for each motion frequency and each experimental condition (A). The VAS profile in accordance with motion frequency was well characterized by the quadric function. Comparison of the peak value (B) and the slope (sharpness) of the quadric function (C) of the quadric function among three experimental conditions. The error bars indicate the standard deviation of the mean value. * Significant difference (p<0.05).

Fig 3 shows the averaged data of the VAS for each motion frequency and each experimental condition. Similar to the results obtained for individual data, the VAS profile in accordance with motion frequency was well characterized by the quadric function. The R^2^ value of the quadric curve was greater than 0.9 under all conditions (Uni: R^2^ = .928, Mirror-: R^2^ = .935, Mirror+: R^2^ = .950). As shown in Fig 3B, the motion frequency at the highest VAS score shows main effect of task (F(2,31) = 3.77, p<0.05), and post hoc analysis revealed significantly higher under Mirror+ than under either of the other two conditions (Uni: 1.17±0.36, Bi_Mirror-: 1.34±0.34, Bi_Mirror+: 1.44±0.28). The steepness of the quadric function did not show statistically significant difference among three conditions.

**Fig 4:**
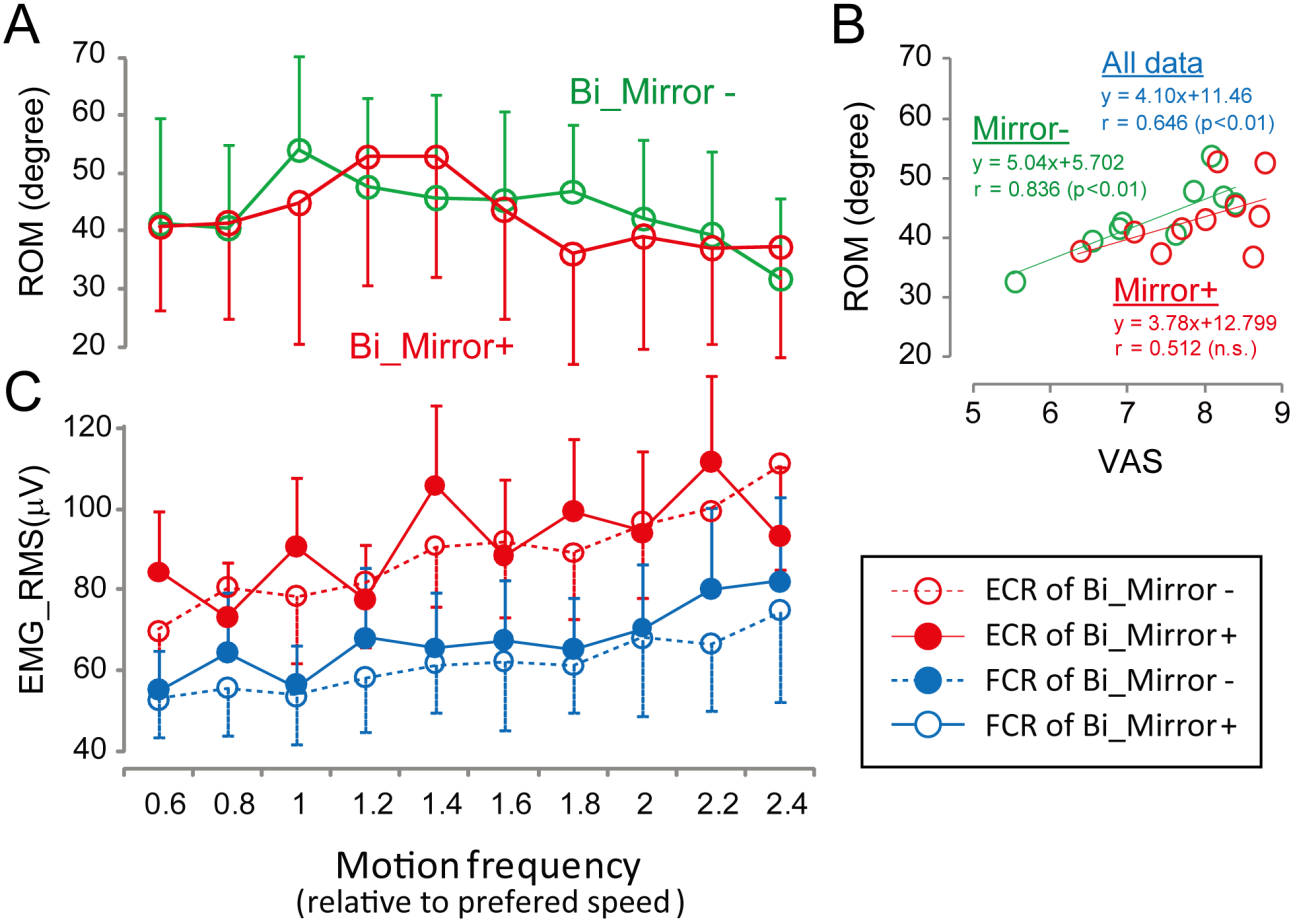
The profile of wrist ROM (A) and EMG amplitudes obtained from ECR and FCR muscles (C) in response to motion frequency. The relationship between VAS and ROM was shown in the right panel (B)

Fig 4A shows the profile of wrist ROM in response to motion frequency. ROM showed an inverted U-shape which is similar to the result of VAS (Fig 3A). Fig 4B shows the relationship between ROM and VAS during Bilateral wrist movement. While there was a significant strong correlation in the Mirror- condition (r = 0.836, p<0.01), the correlation in the Mirror+ condition failed to reach statistical significance (r = 0.512, n.s.). Fig 4C shows profile of the stump muscle EMG amplitude in response to motion frequency. EMG amplitude tended to increase with motion frequency.

**Fig 5:**
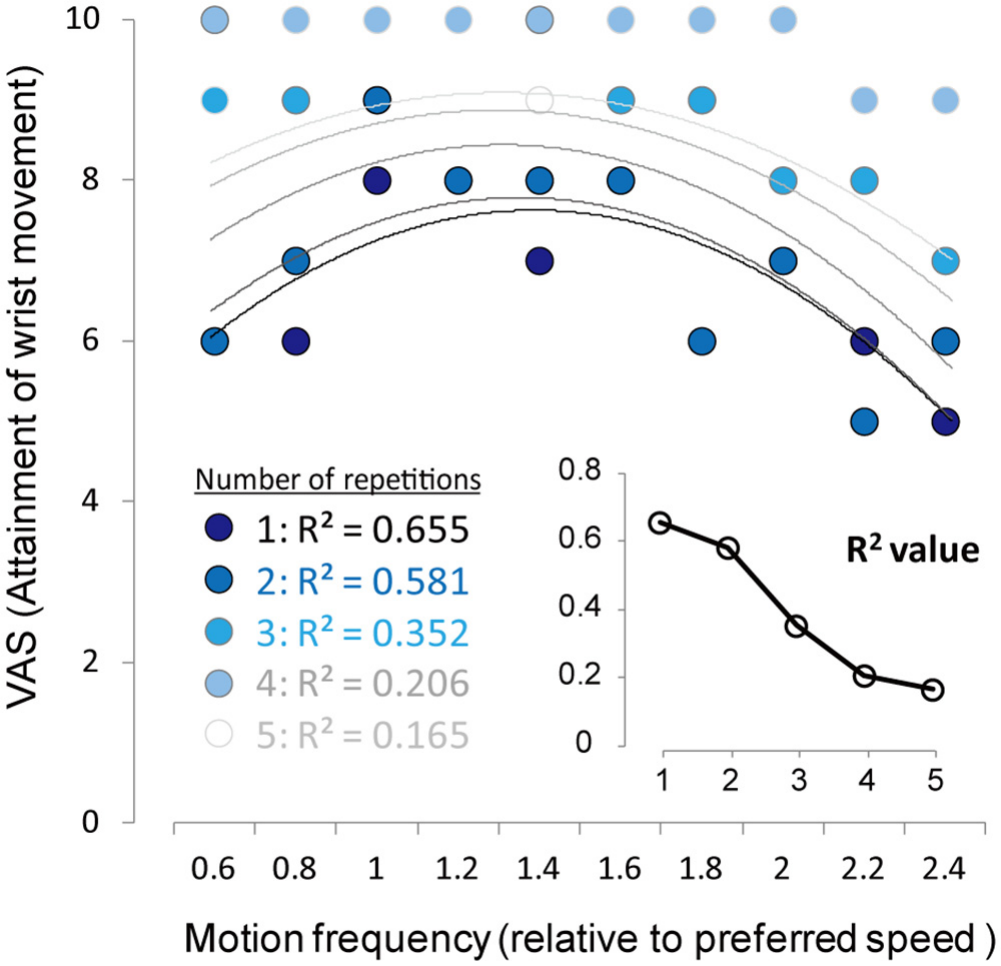
VAS results when Subject L carried out 5 sessions of 10 different bimanual wrist movements. As the imposed line chart clearly indicates, VAS tended to increase and R2 value gradually decreased with the repetition of the session.

Fig 5 shows VAS results when Subject L carried out 5 sessions of 10 different bimanual wrist movements. As the Figure clearly shows, VAS tended to increase and R^2^ value gradually decreased with the repetition of the session.

## Discussion

The purpose of the present study was to propose a novel quantification method for the behavioral aspect of phantom limb phenomenon using psychophysical evaluation. We asked forearm amputees to assess their attainment of phantom limb motion using the VAS. As shown in Fig 2, VASs changed systematically with motion frequency. Although the preferred speed of the phantom limb motion differed among subjects, VAS profiles in accordance with motion frequency were well mathematically fitted by an inverted U-shaped quadric function. Most subjects reported an optimal frequency which enabled them to make phantom limb motion easier, while if the phantom limb motion was slower or faster than the preferred speed, the motion became a little difficult. This comment indicates that the quadric function shows good agreement with subjective feeling.

The previous literature indicates that there is a large inter-individual difference in the modality and extent of phantom limb awareness [17,19]. This can be seen in the movement frequency at the comfortable phantom limb motion recorded during the first screening in the present study. As shown in the rightmost column of Table 1, the preferred speed of the phantom wrist motion ranged from 55 to 80 cycles per minute, and the extent of attainment of the phantom movement ranged from 7 to 10. Such inter-individual difference might prove difficult if one would like to characterize the phantom limb phenomenon. A conventional way to characterize a phantom limb is by describing the patient’s subjective feeling. Almost all outcome measures employed to date in behavioral and clinical studies regarding the extent of awareness and condition of a phantom limb have been subjective or rated on a psychological scale, such as the numeric rating scale (NRS) or VAS [2,3,17]. In the present study, we intended to create a VAS profile as a function in response to changes in motion frequency because such a mathematical fitting can complement the limitations of subjective parameters.

As shown in Table 2, the mathematical fitting explains almost 60–70% of variance for the relationship between VAS and motion frequency. In order to evaluate whether the proposed psychophysical method can work as a probe to differentiate the motion task and correctly reflect the accomplishment of the phantom limb motion, wrist ROM and root mean squared value of stump muscle EMG were also calculated. As shown in Fig 3, ROM showed an inverted U-shape similar to that of VAS, and positive correlation was found between VAS and ROM. While the modulation pattern was not similar to the ROM and VAS, EMG amplitude tended to increase with motion frequency. These results suggest that the proposed method can be useful for identifying task differences. The degree of steepness (identified as the coefficient of the squared term) and shift of the quadric function (motion frequency at maximum VAS) can be regarded as parameters that reflect the characteristics of phantom limb motion. For example, the motion frequency at maximum VAS score might reflect the comfortable (optimal) motion frequency of the phantom limb, and steepness of the quadric function might indicate the affordability of the motion frequency range at which the subject can comfortably perform phantom limb motion. As shown in Fig 3B, the motion frequency at maximum VAS shows statistically significant difference among three experimental conditions, suggesting that not only simultaneous bimanual intact and phantom movement but also mirror-reflected visual feedback would have potential resources to facilitate an attainment of phantom limb movement. Given the fact that the steepness of the quadric function and maximum VAS score did not show statistically significant difference among conditions, shift of preferred speed would be more sensitive parameter for the task-dependent changes of the phantom limb movement. These results strongly suggest that the psychophysical method we attempted in the present study has the potential to characterize task-dependent differences in phantom limb condition, taking into consideration inter-individual differences in the preferred phantom limb motion and extent of awareness.

In the present study, we applied psychometric function based on one trial of each motion frequency. Since the number of data greatly affects the validity of mathematical fitting, one might think that insufficient number of trial would be a sort of methodological limitation. In order to confirm how the number of trials might affect the validity of the mathematical fitting, we asked Patient L to perform 5 trials of each motion frequency under bilateral phantom movement without MVF (Mirror-) on a different day from the main experiment. We found that R^2^ value decreased with the repetition of the movement, a result presumably at least partly explained by habituation to the phantom limb movement. With regard to the standard process to achieve a better mathematical fitting, it is necessary to obtain enough data to confirm data reproducibility. On the other hand, our results suggest that our proposed evaluation approach would effectively work to characterize phantom limb motion even using only one trial. This is an advantage for clinical use in terms of minimizing the total trials and the subject’s effort.

In the present study, while we focused primarily on phantom limb movement, we also asked subjects about the extent of phantom limb pain and about relevant perceptions regarding phantom limb motion after the experiment. Three patients experienced a decrease in phantom limb pain with MVF, and one told us that, when he could not follow the tempo of the metronome due to an increase in movement frequency, the extent of phantom limb numbness also increased. This suggests that the occurrence of phantom limb numbness may be relative to the accomplishment of phantom limb movement. Further investigation is needed to clarify this point.

In conclusion, the present results demonstrate the potential usefulness of psychophysical evaluation to assess phantom limb condition. The proposed method has potential applications in assessing rehabilitation effect. Further study is required to examine more detail mechanisms underlying phantom limb and phantom limb pain.
